# Inequitable access to risk-appropriate neonatal care: evidence from a regionalized perinatal system in Arkansas

**DOI:** 10.3389/frph.2026.1914178

**Published:** 2026-08-14

**Authors:** Md Asif Khan Sharon, Sandra D. Eksioglu, Cari Anne Bogulski, Enrique Gomez Pomar

**Affiliations:** 1 Department of Industrial Engineering, University of Arkansas, Fayetteville, AR, United States; 2 Department of Biomedical Informatics, University of Arkansas for Medical Sciences, NW Campus, Fayetteville, AR, United States; 3 Institute for Community Health Innovation, University of Arkansas for Medical Sciences, Springdale, AR, United States; 4 Department of Pediatrics, University of Arkansas for Medical Sciences, Little Rock, AR, United States

**Keywords:** geographic access to care, health service research, perinatal regionalization, risk-appropriate care, triage alignment

## Abstract

**Objective:**

To evaluate access to risk-appropriate neonatal care in Arkansas and examine relationship among geography, hospital availability, county-level maternity care access, birth triage patterns, neonatal transfers, and neonatal intensive care unit (NICU) utilization.

**Study design:**

Population-based retrospective cohort study of all live births in Arkansas from 2014 to 2023 using linked birth certificate, hospital designation, and county-level maternity care access data. Primary exposures included geographic distance to the nearest risk-appropriate facility, local hospital availability, and insurance status. Outcomes included birth triage status (over-, under-, or risk-appropriate), transfer triage patterns, NICU utilization, and the impact of obstetric unit closures.

**Results:**

Among 358,155 live births, 76.5% occurred at facilities providing a higher level of care than required, 20.1% occurred at risk-appropriate facilities, and 3.3% occurred at facilities providing a lower level of care than required. Under-triage was most common when the nearest risk-appropriate facility was geographically distant (mean distance 51.2 miles). Medicaid-covered births experienced under-triage nearly twice as often as privately insured births (4.31% vs. 2.55%; *p* < 0.001). NICU admission rates varied substantially across county access categories (6.5%–9.3%) despite similar rates of high-acuity births (3.5%–3.9%), consistent with supply-sensitive utilization. Among 6,935 neonatal transfers, 84.3% were directed to facilities providing a higher level of care than required. Of 4,703 transfers to Level IV NICUs, only 3.6% of infants met criteria for Level IV care, and a closer Level III facility was available in 96.1% of cases. Following obstetric unit closures, risk-appropriate births decreased, over-triage increased, and travel distances lengthened.

**Conclusion:**

Access to risk-appropriate neonatal care was shaped more strongly by geography, resource distribution, and referral structure than by measured clinical risk. These findings suggest that regionalized perinatal systems may simultaneously experience inequitable access and supply-sensitive utilization. Strategies including independent transfer triage coordination, preservation of lower-level maternity and nursery capacity, and standardized NICU admission practices may improve alignment between neonatal need and site of care.

## 1 Introduction

Perinatal regionalization is designed to ensure that pregnant women and newborns receive care at hospitals matched to their medical risk level (1–3). This model concentrates high-risk pregnancies and neonatal cases at specialized centers while allowing lower-level hospitals to manage routine and moderate-risk deliveries. A substantial body of evidence shows that this approach improves outcomes, particularly for very preterm and very low-birth-weight infants, who have lower mortality and morbidity when delivered at level III or IV neonatal intensive care units (NICUs) rather than at non-specialty facilities (1, 4).

Critically, the benefits of risk-appropriate care are bidirectional: while under-triage of high-risk infants increases mortality, over-triage of lower-risk infants to higher-level facilities does not appear to confer a survival advantage and may cause harm. A meta-analysis demonstrated that VLBW infants born outside of level III hospitals had significantly higher odds of neonatal death, with an even stronger effect among extremely low-birth-weight infants (5). Conversely, for late preterm and term infants, delivery at hospitals with higher NICU capacity has been associated with increased NICU admission without improved short-term outcomes (7, 8).

In the United States, neonatal intensive care capacity has expanded substantially since the early implementation of regionalized perinatal care, but the effects of this growth have been mixed (4). Although this expansion has coincided with major declines in infant mortality (6), growth in neonatal resources has not consistently aligned with population-level perinatal risk. Recent national studies have shown that increases in NICU capacity have not been accompanied by comparable reductions in newborn mortality, and that higher NICU bed supply is associated with greater admission of late preterm and term infants without improved short-term outcomes (7). Together, these findings suggest that NICU expansion may promote supply-sensitive utilization rather than reflect unmet clinical need (8).

National NICU admission rates have continued to increase despite relatively stable birth volumes, with increases observed across most gestational age, birthweight, and demographic groups. Prior studies have documented geographic variation in NICU utilization and quality of care, and racial and ethnic differences in neonatal care have also been reported nationally, suggesting that structural characteristics of perinatal systems may influence access and use independently of clinical need (7, 9). These patterns raise concern for both overuse of high-level services and inadequate access to appropriate care in underserved areas. Publicly insured families may be especially affected, because Medicaid coverage is disproportionately concentrated in maternity care deserts with limited obstetric and neonatal access (10).

Arkansas is a key example of these challenges. Despite statewide efforts to strengthen perinatal regionalization, access to maternity care has worsened in many areas, particularly in rural counties (11, 12). Since 2019, seven birthing hospitals in Arkansas have closed their obstetric units, further limiting local access and increasing travel distances for delivery and neonatal care (13–15). How these structural constraints influence risk-appropriate placement at birth and during inter-facility transfer remains incompletely understood (16).

Using linked birth certificate data from 2014 through 2023, this study had three objectives: to evaluate access to risk-appropriate neonatal care and triage at birth in Arkansas, including variation by insurance status; to assess how geographic proximity, local hospital availability, obstetric unit closures, and county-level maternity care access were related to NICU utilization; and to evaluate neonatal transfer patterns. This study focuses on triage patterns and access rather than neonatal outcomes. Triage alignment is a system-level measure of whether regionalized care places infants in facilities matched to their measured care needs and provides a foundation for subsequent outcome evaluation

## 2 Methods

We conducted a population-based retrospective cohort study of all live births occurring in Arkansas from January 1, 2014, through December 31, 2023. Birth certificate records from the Arkansas All-Payer Claims Database were linked to county-level maternity care access classifications from the March of Dimes (16). We constructed a facility registry using the 2016 Arkansas Department of Health's perinatal regionalization list, the Arkansas Center for Health Improvement (ACHI) hospital roster, and the U.S. Maternal Health Portal, verifying NICU levels (I–IV) via hospital websites when necessary (14, 17, 18).

The required neonatal care level was assigned using birthweight, gestational age, and clinical complications per American Academy of Pediatrics (AAP) guidelines (19, 20). Infants with major surgical anomalies were assigned Level IV care. Level III care was assigned to infants who were very preterm (<32 weeks' gestation), very low birth weight (<1,500 g), or critically ill, including those requiring prolonged mechanical ventilation, surfactant therapy, seizure management, or experiencing severe 5 min Apgar depression. Level II care was assigned to moderate preterm infants (32–36 weeks' gestation), low-birth-weight infants (1,500–2,499 g), or infants with moderate illness requiring brief ventilation or with 5 min Apgar scores of 4–6. All remaining uncomplicated births were classified as requiring Level I care.

Triage alignment was assessed by comparing each infant's required level of neonatal care, assigned using AAP-based clinical criteria, with the neonatal level of the delivery hospital (for births) or receiving hospital (for transfers). Births or transfers were classified as risk-appropriate when the hospital level matched the infant's required level of care, over-triaged when care occurred at a hospital providing a higher level of neonatal services than required, and under-triaged when care occurred at a hospital providing a lower level of neonatal services than required.

Travel distance was estimated as geodesic (straight-line) distance in miles from the maternal county centroid to the delivery hospital for births and from the birth hospital to the receiving hospital for transfers, and local availability was assessed by counting facilities within 50, 100, and 150-mile bands (21). Of 358,155 births, 356,017 (99.4%) were linked to county access data for analyses of NICU utilization and transfers; triage and distance analyses used 347,289 births with complete facility and care-need data. We conducted pre–post analyses of five obstetric unit closures comparing birth and transfer outcomes; inferential analyses were limited to counties with sufficient post-closure follow-up. The closure analysis focused on obstetric unit closures; NICU closures or changes in NICU designation were not separately validated across the full study period.

Categorical outcomes across access groups, payment type and pre-post closure periods were compared using ꭓ^2^ tests, with Fisher's exact tests used when cell counts were small. Effect sizes were reported using Cramér's V. Wilson 95% confidence intervals were calculated for estimated proportions. Differences in median travel distances were evaluated using Mann–Whitney *U*-tests. Temporal trends were assessed at the county–year level using Kruskal–Wallis tests, restricted to county-years with ≥50 births or ≥5 transfers to ensure stability. County-level correlations between access metrics (median distance to the nearest risk-appropriate facility; average number of risk-appropriate facilities within 50 miles), birth volume, and the risk-appropriate birth rate were assessed using Pearson and Spearman correlation tests. The county-level analyses were descriptive and correlational and were not designed to estimate independent effects after adjustment for potential confounders. All statistical tests were two-sided, with statistical significance defined as *α* = 0.05. Analyses were performed in Python (v3.7.6) using SciPy, statsmodels and NumPy; figures were generated with Matplotlib and Seaborn. Additional methodological details are provided in the Supplement.

## 3 Results

### 3.1 Study population

From January 1, 2014, through December 31, 2023, there were 358,155 live births in Arkansas. March of Dimes county-level maternity care access varied substantially across the state, with counties classified as having full access, moderate access, low access, or no access to maternity care. Neonatal transfers occurred in 7,404 births (2.1%) during the study period, and 6,935 of those cases had valid facility data and were included in the transfer analysis.

### 3.2 Geographic distribution of facilities and maternity-care access

**Figure 1:**
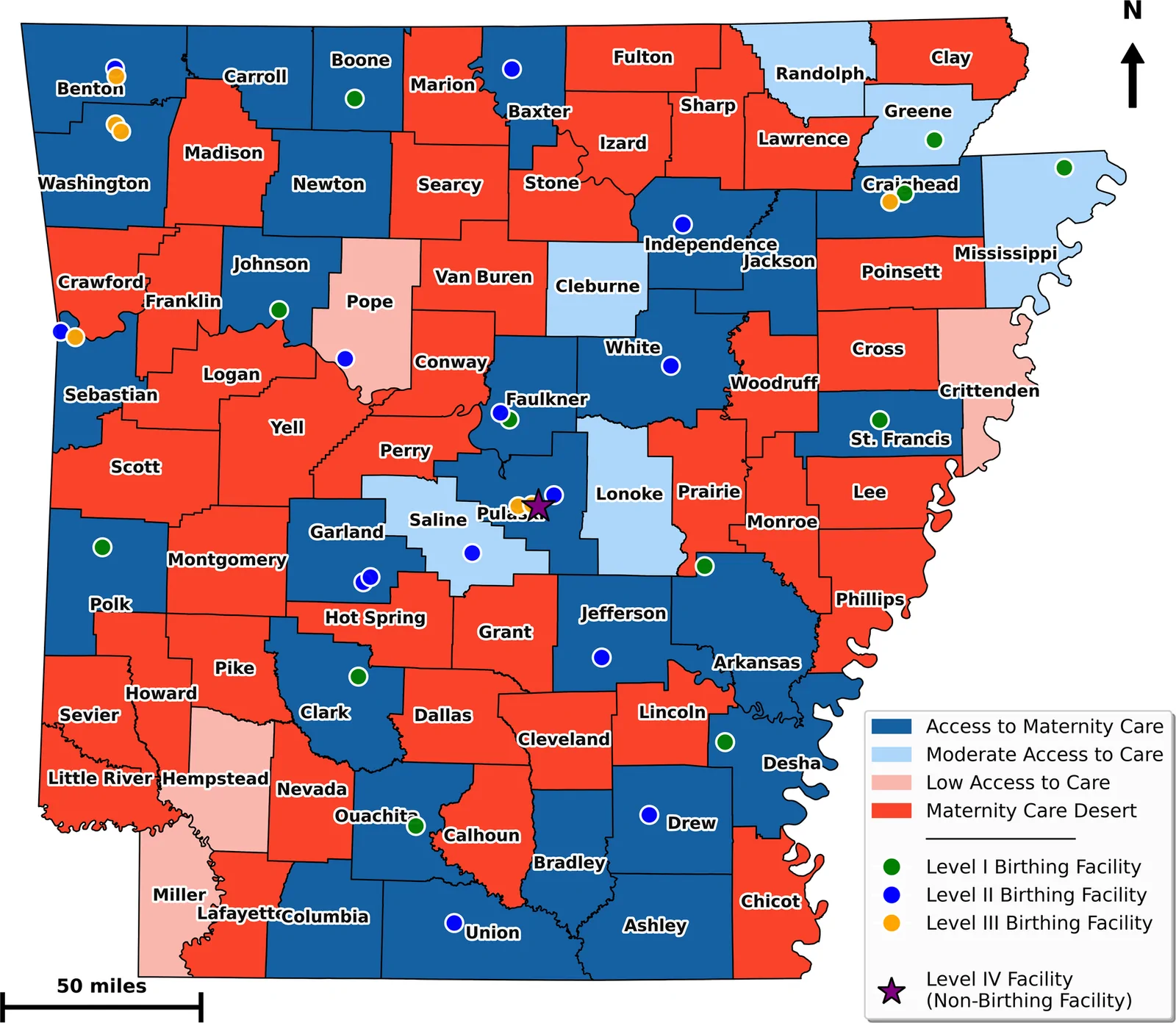
Geographic distribution of maternity-care access and neonatal facilities in Arkansas. County shading indicates maternity-care access classification. Circles represent Level I–III birthing hospitals. The star represents the state's only Level IV neonatal referral center, which does not provide delivery services.

The geographic distribution of perinatal care facilities was highly uneven across Arkansas. Higher-level birthing hospitals (Levels III and IV) and the state's only Level IV non-birthing children's hospital were concentrated in central and northwestern Arkansas, whereas southern and eastern regions had substantially fewer facilities and more limited access to specialized neonatal services (Figure 1). Consistent with this pattern, many counties in these regions were classified as low-access areas or maternity care deserts, highlighting significant geographic disparities in access to risk-appropriate maternal and neonatal care (Figure 1).

### 3.3 Birth triage and access to risk-appropriate care

Across all years, 20.1% of infants were delivered at risk-appropriate facilities, 76.5% were over-triaged, and 3.3% were under-triaged (Table 1). Risk-appropriate birth rates declined from 21.3% in 2014 to 19.0% in 2023, while over-triage increased from 75.2% to 77.9%. Under-triage remained stable at approximately 3.3% throughout the study period.

**Table 1:** Annual triage rates at birth and neonatal transfers in Arkansas, 2014–2023.

Year	Birth triage				Neonatal transfer triage			
	*N* (Total)	RA (%)	OT (%)	UT (%)	*N* (Total)	RA (%)	OT (%)	UT (%)
2014	36,248	21.3	75.2	3.5	727	20.8	75.8	3.4
2015	36,398	21.7	74.8	3.4	761	16.4	81.2	2.4
2016	35,749	21.2	75.5	3.3	801	16.6	79.2	4.2
2017	35,073	20.3	76.2	3.4	790	15.4	82.9	1.6
2018	34,934	20.2	76.6	3.3	734	16.5	79.8	3.7
2019	34,558	20.4	76.2	3.4	774	14.5	83.9	1.7
2020	33,609	19.9	76.7	3.5	703	12.4	86.3	1.3
2021	34,313	19.1	77.7	3.3	700	13.6	83.6	2.9
2022	33,608	18.3	78.5	3.2	667	12.9	84.4	2.7
2023	32,799	19	77.9	3.1	747	10.2	86.6	3.2
Avg.	34,729	20.1	76.5	3.3	740	14.9	82.4	2.7

RA, Risk-Appropriate; OT, Over-Triage; UT, Under-Triage. Birth triage averages are weighted by annual volume.

Hospital availability differed by triage category (Table 2). Risk-appropriate births occurred in areas with a relatively balanced facility mix, with a mean of 5.3 appropriate and 5.2 higher-level hospitals within 100 miles. Over-triage was associated with saturation of specialized resources, with higher-level facilities outnumbering appropriate options (7.4 vs. 6.3 within 100 miles). Under-triage occurred in areas where lower-level facilities predominated and specialized centers were limited (11.2 lower-level vs. 1.7 appropriate facilities within 100 miles).

**Table 2:** Average number of hospitals within 50, 100, and 150 miles, by triage outcome (*n* = 347,298) and hospital level, using two reference points: (A) mother's county of residence; and (B) birth facility for transfers.

Source	Outcome	Dist.	Average number of Hospitals		
			RA	Higher	Lower
(A) Birth: mother's county of residence	RA (*n* = 69,986)	≤50	1.6	1.5	1.9
		≤100	5.3	5.2	6.2
		≤150	9.4	9.4	10.9
	Over-Triage (*n* = 265,666)	≤50	1.9	2.1	0.9
		≤100	6.3	7.4	2.9
		≤150	11.1	13.3	5.2
	Under-Triage (*n* = 11,637)	≤50	1.1	0.5	3.4
		≤100	3.8	1.7	11.2
		≤150	6.7	3.2	19.9
(B) Transfer: birth facility	RA (*n* = 1,092)	≤50	0.8	0.2	3.3
		≤100	2.5	0.8	12.6
		≤150	4.6	1.4	21.7
	Over-Triage (*n* = 5,820)	≤50	1.4	1.6	1.5
		≤100	5	5.1	5.9
		≤150	8.8	9.1	10.1
	Under-Triage (*n* = 23)	≤50	0.4	0	3.4
		≤100	1.6	0.4	11.6
		≤150	3.2	0.9	20.5

RA, Risk-Appropriate.

**Figure 2:**
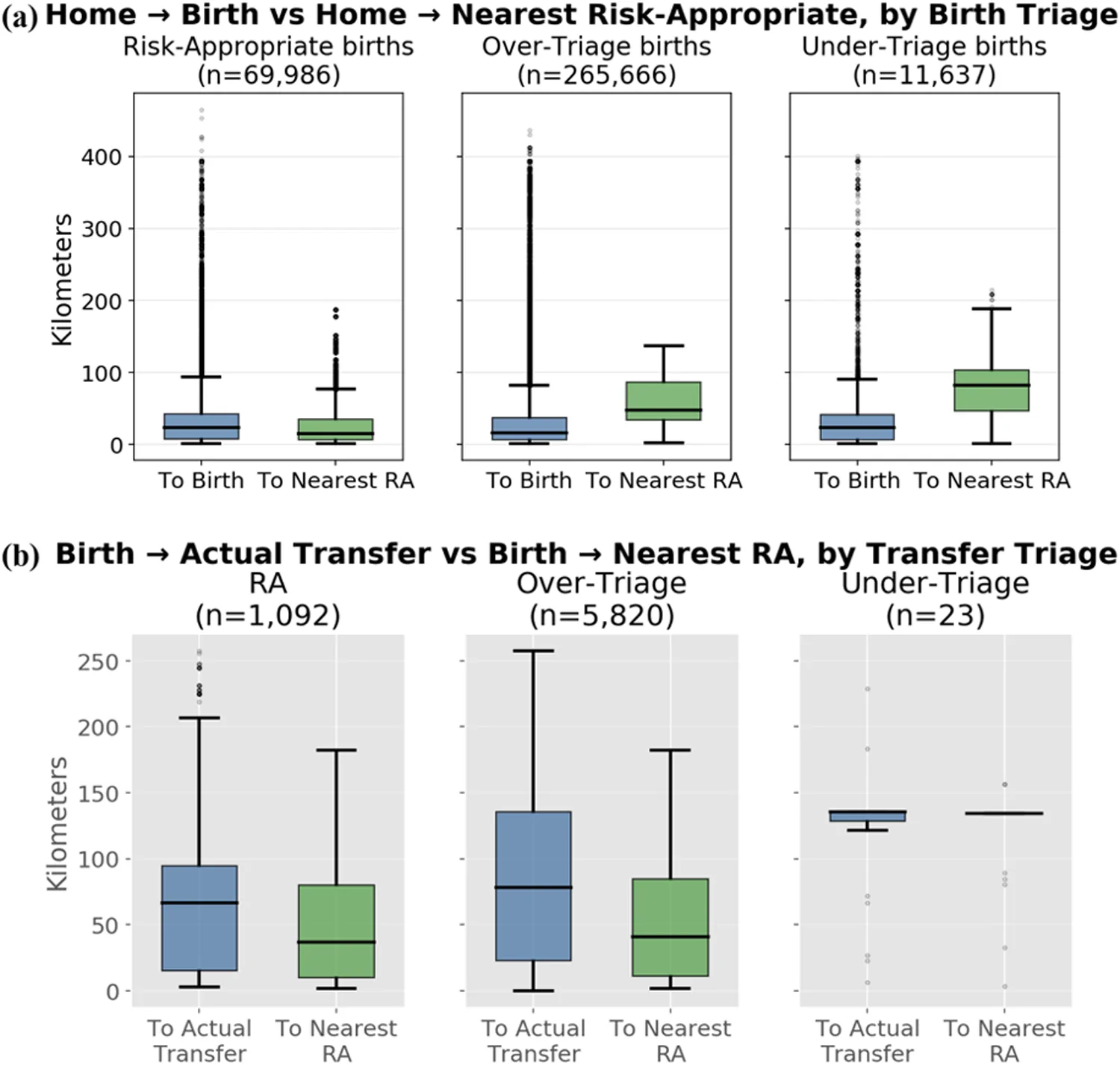
Geodesic distance by triage alignment at birth and transfer. **(a)** Distance from maternal residence to the actual birth facility and to the nearest risk-appropriate birth facility, stratified by birth triage. **(b)** Distance from the birth facility to the actual transfer destination and to the nearest risk-appropriate receiving facility, stratified by transfer triage. Boxes indicate the interquartile range, center lines indicate medians, whiskers indicate the non-outlier range, and points indicate outliers.

At the county level, there was a moderate inverse correlation between county-level risk-appropriate birth rate and median distance to the nearest risk-appropriate facility (Spearman *ρ* = −0.56, *p* < .001; Table 3). For risk-appropriate births, the median travel distance to the delivery hospital was 15.1 miles. For over-triaged births, the median distance to the higher-level hospital (10.2 miles) was significantly shorter than the distance to the nearest appropriate facility (29.9 miles; *p* < 0.001). Under-triage occurred when the nearest risk-appropriate hospital was on average 51.2 miles away, compared to a median of 14.6 miles to the lower-level hospital where delivery occurred (Figure 2a).

**Table 3:** Descriptive comparisons of county-level access, triage, NICU utilization, and correlation summaries, 2014–2023.

Panel A. Descriptive comparisons by county-level maternity care access category						
Outcome	County Access Level				Overall *χ*² test	
	Full	Moderate	Low	Desert	χ2(df = 3)	*p* value
Birth Facility						
RA (%)	17.28 [17.13, 17.44]	26.76 [26.31, 27.22]	33.71 [32.83, 34.60]	24.80 [24.46, 25.14]	4,289.5	<0.001
OT (%)	79.96 [79.80, 80.12]	68.34 [67.86, 68.81]	60.69 [59.77, 61.61]	70.89 [70.54, 71.25]	5,544.3	<0.001
UT (%)	2.76 [2.69, 2.82]	4.90 [4.68, 5.12]	5.60 [5.18, 6.04]	4.31 [4.16, 4.47]	878.7	<0.001
NICU Uti (%)	9.31 [9.19, 9.42]	8.24 [7.96, 8.52]	6.51 [6.07, 6.98]	8.98 [8.76, 9.20]	138.5	<0.001
H.Acuity (%)	3.93 [3.85, 4.00]	3.49 [3.31, 3.68]	3.61 [3.28, 3.97]	3.59 [3.45, 3.74]	29.2	<0.001
Transfer Facility						
Trnsf rt (%)	1.85 [1.79, 1.90]	2.53 [2.37, 2.69]	3.21 [2.90, 3.55]	2.41 [2.29, 2.53]	206	<0.001
OT (%)	81.99 [80.82, 83.10]	84.80 [82.34, 86.97]	90.93 [87.48, 93.51]	89.10 [87.43, 90.57]	55.9	<0.001
RA (%)	17.67 [16.57, 18.83]	14.66 [12.52, 17.09]	9.07 [6.49, 12.52]	10.50 [9.06, 12.15]	56.6	<0.001

Panel B. County-level Pearson and Spearman correlations					
Relationship	*n*	Pearson r	*p* value	Spearman *ρ*	*p* value
Risk-appropriate birth rate vs. median distance to RA	75	−0.508	<0.001	−0.555	<0.001
Risk-appropriate birth rate vs. birth volume	81	−0.317	0.004	−0.328	0.003
Median distance to RA vs. RA facilities within 50 mi	75	−0.548	<0.001	−0.452	<0.001
Risk-appropriate birth rate vs. RA facilities within 50 mi	81	0.184	0.1	0.155	0.166

Panel A presents descriptive comparisons across county-level maternity care access categories. At-birth outcomes use all live births as the denominator. Transfer rate uses all live births as the denominator, while transfer-triage composition rows use transferred infants as the denominator. Values are rates with Wilson 95% confidence intervals. Overall χ² tests are 2 × 4 tests comparing each outcome across county access levels.

Panel B presents county-level Pearson and Spearman correlation coefficients between access metrics, birth volume, and risk-appropriate birth rates. Access levels: Full, Full Access to Maternity Care; Desert, Maternity Care Desert; RA, Risk-Appropriate; OT, Over-Triage; UT, Under-Triage; NICU Uti, NICU utilization; H.Acuity, high-acuity birth rate requiring Level III/IV care; Trnsf rt, transfer rate.

Birth triage varied by county-level maternity care access (Table 3). Counties with full access exhibited the highest over-triage rate (79.96% [95% CI, 79.80–80.12]) and the lowest under-triage rate (2.76% [95% CI, 2.69–2.82]). Low-access counties showed the opposite pattern, with over-triage at 60.69% [95% CI, 59.77–61.61] and under-triage at 5.60% [95% CI, 5.18–6.04].

Triage patterns differed significantly by payment source (*p* < 0.001). Over-triage was more frequent among births with private insurance (80.79% [95% CI, 80.60–80.98]) than those covered by Medicaid (71.25% [95% CI, 71.02–71.48]). Medicaid-covered births experienced a significantly higher rate of under-triage (4.31% [95% CI, 4.21–4.41]), nearly twice the rate of those with private insurance (2.55% [95% CI, 2.47–2.62]).

Higher county birth volume was weakly to moderately inversely correlated with the county-level risk-appropriate birth rate (Spearman *ρ* = −0.33, *p* = 0.003; Table 3). In high-volume counties such as Washington (31,436 births) and Benton (36,443 births), risk-appropriate rates were 4.9% and 6.2%, respectively, where the median distance to a risk-appropriate facility was nearly 60 miles. Even in Pulaski County, which has more nearby facilities (2.96 within 50 miles) and a shorter travel distance (21.9 miles), the risk-appropriate rate was only 8.6%.

Transfer over-triage also varied significantly by payment source (*p* = .003, Cramér's *V* = 0.047). Over-triage was highest among privately insured transfers (86.2% [95% CI, 84.9–87.4]) and Medicaid-covered transfers (84.9% [95% CI, 83.7–86.0]), compared with self-pay transfers (82.5% [95% CI, 77.5–86.6]) and transfers with other payment methods (75.3% [95% CI, 64.4–83.8]). This pattern contrasts with birth triage, where Medicaid-covered infants experienced the highest rates of under-triage.

### 3.4 NICU utilization

NICU admissions occurred in 9.1% (32,443 of 358,155) of births statewide. Admission rates were highest in full-access counties (9.31% [95% CI, 9.19–9.42]) and maternity care deserts (8.98% [95% CI, 8.76–9.20]), and lowest in low-access counties (6.51% [95% CI, 6.07–6.98]) (Table 3). In contrast, the rate of high-acuity births (requiring Level III or IV care) was relatively uniform across all access categories, ranging from 3.49% to 3.93%. Despite similar rates of high-acuity births across access categories, NICU admission rates varied by nearly three percentage points, suggesting that variation in NICU use may reflect differences in local NICU capacity, admission thresholds, and hospital practice patterns in addition to clinical need.

### 3.5 Neonatal transfers

Neonatal transfers were classified as over-triaged in 82.4% of cases, risk-appropriate in 14.9%, and under-triaged in 2.7% of cases. Over-triage among transfers increased over the study period, rising from 75.8% in 2014 to 86.6% in 2023, while risk-appropriate transfers declined from 20.8% to 10.2% (Table 1).

Transfer rates also varied by county access level. Low-access counties had the highest transfer rate (3.21% [95% CI, 2.90–3.55]), whereas full-access counties had the lowest (1.85% [95% CI, 1.79–1.90]). Risk-appropriate transfers were most common in full-access counties (17.67% [95% CI, 16.57–18.83]) and least common in low-access counties (9.07% [95% CI, 6.49–12.52]).

The median distance to the transfer destination was 48.1 miles, compared with 26.3 miles to the nearest risk-appropriate hospital. For over-triage transfers (*n* = 5,820; 83.9%), the median distance difference between the actual destination and the nearest risk-appropriate hospital was 10.8 miles. Risk-appropriate transfers (*n* = 1,092; 15.7%) were generally close to the nearest appropriate option, with a median distance difference of 1.3 miles (Figure 2b).

Infants transferred to higher-level facilities differed clinically by transfer triage category. Over-triaged transfers involved infants with a higher mean birth weight (2,778 g) and greater mean gestational age (36 weeks) than infants transferred appropriately (2,119 g and 33 weeks; *p* < 0.001 for both).

Among 4,703 transfers to Level IV NICUs, only 3.6% of infants required Level IV care. Most transferred infants required Level I or II care (65.1%) or Level III care (31.3%). The transferred population was predominantly term (55.5%), with a mean gestational age of 35.8 weeks and mean birth weight of 2,750 g. Most transferred newborns had a normal 5 min Apgar score (65.9%), and only 3.6% had major surgical anomalies, a Level IV indicator. In 96.1% of Level IV transfers, a Level III facility was located closer to the birth hospital than the Level IV destination.

Hospital availability around the birth facility varied by transfer triage category (Table 2). For risk-appropriate transfers, the local supply favored the correct level of care, with 2.5 risk-appropriate vs. 0.8 higher-level facilities within 100 miles. For over-triage transfers, the surrounding supply within 100 miles was nearly identical between risk-appropriate and higher-level facilities (5.0 vs. 5.1). Under-triage transfers occurred in the most constrained settings, with almost no risk-appropriate or higher-level facilities within 50 miles.

### 3.6 Impact of obstetric unit closures

During the study period, five Arkansas hospitals closed obstetric units, including four rural Level I hospitals and one urban Level II hospital. In rural counties, loss of local Level I capacity reduced risk-appropriate births and increased over-triage. In Columbia County (Magnolia), risk-appropriate delivery fell from 58.6% to 20.5% and over-triage rose from 29.7% to 73.8% (*p* < 0.001), with local Level I deliveries declining from 66.6% to 13.4% and Level II utilization increasing from 30.2% to 82.1%. In Phillips County (Helena), over-triage also increased (*p* = 0.006), although the effect was moderated by nearby Level I facilities.

In urban Pulaski County, closure of a Level II unit intensified over-triage (*p* < 0.001), with Level I and II infants increasingly delivered at Level III centers. Median travel distances rose from 2.5 to 32.6 miles in Columbia County and from 14.6 to 45.5 miles in Phillips County (both *p* < 0.001). Neonatal transfer patterns changed little after obstetric closures, as infants requiring transfer were already being routed to Level III–IV centers. In Pulaski County, transfer rates rose modestly from 1.0% to 1.5% (*p* < 0.001), while risk-appropriate transfers declined from 21.6% to 14.7%.

## 4 Discussion

In this population-based study of all live births in Arkansas over a 10-year period, fewer than one in five infants were born at risk-appropriate facilities, and over-triage predominated at both birth and transfer. These patterns were stable over time and appeared more closely related to geographic access to care than to variation in measured neonatal risk. The findings suggest that regionalized perinatal systems are being shaped as much by where facilities are located as by the clinical needs of infants.

### 4.1 Supply-sensitive utilization: NICU capacity drives use beyond clinical need

The relatively uniform distribution of high-acuity births across counties suggests that geographic differences in NICU utilization are unlikely to reflect major differences in underlying population risk alone. Because rates of high-acuity births were similar across county access categories, the observed variation in NICU admission rates may reflect differences in local NICU capacity, admission thresholds, referral practices, and hospital practice patterns in addition to clinical need. Instead, these findings are consistent with supply-sensitive utilization, in which availability of specialized resources is associated with patterns of care delivery. In practical terms, this pattern is compatible with greater NICU use among late preterm and term infants in settings where NICU capacity is more readily available (22, 23).

Arkansas provides a useful illustration of this pattern. High-volume counties with more advanced facilities had some of the lowest rates of risk-appropriate birth, suggesting that lower-risk infants were often delivered at higher-level centers where lower-level alternatives were less available or less accessible. This “birth volume paradox” may reflect structural misalignment between facility distribution and population risk rather than more efficient regionalization (8, 24). Because NICU admission was measured using birth certificate data, which likely undercount actual admissions, the magnitude of supply-sensitive utilization may also be underestimated (25).

Although the present study was not designed to assess neonatal outcomes, the triage patterns observed here should be interpreted in light of a growing body of evidence suggesting that both under-triage and over-triage carry clinical consequences. For high-risk infants, the case for risk-appropriate placement is well established: a meta-analysis of 41 studies showed that VLBW infants born outside level III hospitals had significantly higher mortality (adjusted OR, 1.62; 95% CI, 1.44–1.83), and an umbrella review confirmed a pooled effect favoring regionalized care (OR, 1.60; 95% CI, 1.33–1.92) (5, 26). For lower-risk infants, however, the evidence points in the opposite direction. Goodman et al. found that among late preterm newborns in Texas Medicaid, birth at the highest-capacity hospitals was associated with a 17% higher risk of NICU admission (aRR, 1.17; 95% CI, 1.01–1.33) without improvement in 30-day outcomes (8). Gasper et al. demonstrated that three decades of growth in regional NICU capacity were not associated with reductions in newborn mortality (7). Beyond the absence of outcome benefit, unnecessary NICU admission has been associated with decreased breastfeeding continuation among late preterm infants (APR, 0.86; 95% CI, 0.76–0.99) and reduced full breastfeeding at discharge among near-term infants (aOR, 0.40; 95% CI, 0.34–0.47) (27, 28). Unnecessary admissions are also costly, stressful to families, and may increase the risk of hospital-acquired morbidities (9).

While our data do not include outcome measures to directly confirm these relationships in Arkansas, the scale of over-triage documented here — affecting more than three-quarters of all births and over 80% of transfers — suggests that the potential for avoidable harm and excess cost is substantial. Future studies linking these triage patterns to neonatal outcomes, breastfeeding rates, and length of stay would help quantify the clinical consequences of the structural misalignment identified in this analysis.

### 4.2 Geographic barriers and structural inequities in triage

Under-triage occurred mainly when the nearest risk-appropriate facility was far away, strongly suggesting limited access rather than parental choice or clinical necessity. The inverse relationship between distance to a risk-appropriate facility and risk-appropriate birth rates reinforces how geography shapes care pathways. Families often delivered at the nearest available hospital, even when that hospital did not match the infant's level of need.

### 4.3 Obstetric closures disrupt triage at birth, not during transfer

Obstetric unit closures disrupted risk-appropriate placement at birth, particularly in rural counties. Loss of local Level I capacity shifted deliveries toward higher-acuity facilities and sharply increased travel distances, showing how fragile local access can be. In contrast, neonatal transfer patterns changed little after closures, implying that the main disruption occurred at the point of birth rather than during postnatal referral.

This distinction matters because it suggests that preserving local delivery capacity may be more effective than focusing only on transfer systems. Even when transfer pathways remained relatively stable, closure of nearby obstetric units forced families into different delivery patterns and longer travel burdens. In that sense, the closure data reinforce the broader finding that availability of local facilities is a primary driver of risk-appropriate care.

### 4.4 Transfer over-triage

Transfers were frequently directed to facilities with a higher level of care than required, and this pattern intensified over time. Among transfers to Level IV NICUs, only a small minority of infants actually required Level IV care, while most required Level I, II, or III services. The fact that a Level III facility was closer in nearly all Level IV transfers indicates that destination choice was often driven by system structure rather than proximity or acuity alone (9, 24).

A likely contributor to this pattern is the organization of neonatal transport in Arkansas, where the state's only Level IV NICU also serves as the principal neonatal referral center. Because Level IV NICUs often coordinate neonatal transport regionally, a transport system closely linked to a single highest-level receiving hospital may encourage default routing to that center even when lower-level alternatives are geographically closer (29). Although some transfers classified as over-triaged may have been clinically appropriate because of evolving illness, diagnostic uncertainty, or need for subspecialty evaluation not captured in birth certificate data, the overall pattern is consistent with structural concentration of transfer flow at the state's highest-level center.

### 4.5 Insurance status and inequitable access to risk-appropriate care

The insurance-related disparities observed in this study reflect a broader pattern in which Medicaid coverage is associated with structural disadvantages in access to risk-appropriate perinatal care. In our data, Medicaid-covered births experienced under-triage nearly twice as often as privately insured births (4.31% vs. 2.55%; *p* < 0.001), while privately insured births were more frequently over-triaged (80.79% vs. 71.25%). This divergence suggests that insurance status influences not just whether infants receive care, but where they receive it. These findings are consistent with prior evidence that rural Medicaid beneficiaries are less likely to deliver at nonlocal, higher-acuity hospitals even after controlling for clinical complications (aOR 0.76; 95% CI, 0.68–0.86), implying a potential access barrier specific to this population (30). The geographic concentration of Medicaid-covered births in areas with limited facility options likely contributes to this pattern: nationally, Medicaid finances 43% of all births but 50%–60% of rural births, and maternity care deserts disproportionately affect low-income and publicly insured communities (31, 32).

In Arkansas, where approximately 72% of births are Medicaid-financed, this overlap is likely substantial. Kozhimannil et al. found that after controlling for clinical complications, rural Medicaid beneficiaries were actually less likely to give birth at nonlocal hospitals than privately insured women, suggesting a potential access barrier rather than a preference; Medicaid recipients may be constrained to deliver locally even when their clinical needs warrant a higher-level facility (30). Thus, the higher under-triage rate among Medicaid-covered births observed in this study may partly reflect geographic concentration of publicly insured families in areas with limited access to risk-appropriate facilities rather than an independent effect of insurance status.

The transfer data add a further dimension. While Medicaid-covered infants experienced the highest rates of under-triage at birth, transfer over-triage was similarly high across insurance categories (84.9% for Medicaid vs. 86.2% for private insurance), suggesting that once a transfer is initiated, the destination is driven more by system structure — particularly the centralized role of the state's only Level IV NICU — than by payer status. This contrasts with earlier literature suggesting that uninsured and Medicaid-covered neonates were more likely to be transferred than commercially insured infants, a phenomenon attributed to financially motivated transfers (33). In our data, the insurance-related disparity appears to operate primarily at the point of birth rather than during transfer, consistent with the hypothesis that Medicaid-covered families face greater geographic and structural barriers to reaching risk-appropriate facilities for delivery but are subject to the same referral pathways once postnatal transfer is initiated.

These findings have important policy implications. The disproportionate under-triage of Medicaid-covered births may reflect not only geographic barriers but also the downstream effects of lower Medicaid reimbursement rates, which contribute to provider shortages and obstetric unit closures in rural areas (34, 35). Addressing insurance-related disparities in risk-appropriate care will likely require both demand-side interventions — such as expanding Medicaid eligibility and extending postpartum coverage — and supply-side strategies, including increasing Medicaid reimbursement rates for obstetric and neonatal services to sustain lower-level maternity capacity in underserved areas (35, 36).

### 4.6 Economic consequences of over-triage

The economic implications of these triage patterns are substantial. NICUs account for over 35% of all pediatric in-hospital costs in the United States, with median total costs of $6,267 for term infants admitted for non-critical reasons (37). Vance et al. reported that median standardized NICU hospital costs rose approximately 20% between 2017 and 2022, suggesting that the economic burden of unnecessary utilization is increasing (38). Neonatal transport adds further costs and long-distance transport further compounds family burden through separation of the maternal–infant dyad, which can disrupt bonding and breastfeeding (39, 40).

Using conservative published cost estimates, these 5,820 over-triaged transfers may represent substantial excess expenditure, estimated at $44–67 million over 10 years. This estimate does not account for the far larger population of over-triaged births (*n* = 265,666) in which delivery at higher-level facilities may have led to NICU admissions that would not have occurred at a risk-appropriate facility (23).

### 4.7 Policy implications: A three-tier strategy for system reconfiguration

These findings support three practical strategies for system reconfiguration. First, neonatal transfer triage should be centralized and independent of the receiving facility to reduce destination bias and improve alignment with acuity. Second, lower-level nursery capacity should be preserved and strengthened in underserved areas so more infants can receive care closer to home (9). Sustaining this capacity will require addressing the financial pressures driving closures, including inadequate Medicaid reimbursement for obstetric and neonatal services (34, 35). Third, admission and transfer thresholds should be standardized to reduce variation in NICU use across hospitals and counties (41).

Telemedicine and back-transport policies could also help support these goals. Telemedicine-assisted neonatal consultation represents an additional strategy for reducing unnecessary transfers and therefore reducing costs (12, 42, 43). Back transported infants are discharged home sooner, have lower recovery charges per infant and have low transfer failures (9.1%) (44–46). Despite this evidence, very few infants are transferred back with significant regional variation and only a few states have established transport policies (16, 46). Together, they offer a feasible way to make regionalized systems more efficient without undermining access for infants who truly need specialty care.

### 4.8 Limitations

This study has several limitations. Birth certificate data may incompletely capture NICU admissions, particularly among term infants; therefore, NICU utilization and supply-sensitive use may be underestimated (25). Geodesic distance is an imperfect proxy for travel burden. The analyses were descriptive and correlational and were not designed to estimate independent effects after adjustment for potential confounders, and the study does not directly measure neonatal outcomes. Because maternal and neonatal care needs are interdependent, some births or neonatal transfers classified as over-triaged may have been influenced by maternal indications for delivery or transfer to a higher-level center, which could not be evaluated in these data. Additionally, race and ethnicity data were not incorporated in these analyses, which limits characterization of equity-related disparities. Similarly, the association between Medicaid coverage and higher under-triage rates may be confounded by geographic distance, as Medicaid-covered births are disproportionately concentrated in rural areas with greater travel distances to risk-appropriate facilities; future studies should examine these dimensions. Even so, the statewide scope and consistency of findings across multiple outcomes strengthen the overall inference. These findings may not be generalizable to states with different geographic, demographic, or health system characteristics.

## 5 Conclusion

This population-based study demonstrates that access to risk-appropriate neonatal care in Arkansas is shaped more by the geography and organization of perinatal resources than by infant clinical risk. Three interconnected patterns emerged: over-triage predominated at both birth and transfer, NICU utilization varied independently of population-level acuity, and Medicaid-covered infants bore a disproportionate burden of under-triage at birth. Together, these findings suggest that regionalized perinatal systems can simultaneously produce inequitable access for high-risk infants in underserved areas and supply-sensitive overuse of specialized resources in well-resourced areas.

Addressing these dual challenges will require coordinated action across multiple levels. The most immediate priorities include establishing acuity-based transfer triage that is independent of any single receiving facility, standardizing NICU admission thresholds to reduce supply-sensitive utilization and sustaining lower-level maternity and nursery capacity in underserved areas through targeted financial support — including adequate Medicaid reimbursement for obstetric and neonatal services. Longer-term strategies should include telemedicine-assisted neonatal consultation to reduce unnecessary transfers, formal back-transport protocols, and regulatory oversight of NICU capacity growth to align resources with population need.

Future research should link triage patterns to neonatal outcomes (including mortality, morbidity, and breastfeeding) and incorporate race/ethnicity-stratified analyses to determine whether triage misalignment compounds existing disparities. Cost-effectiveness evaluations of system reconfiguration strategies would provide the evidence base needed to justify implementation at scale.

## Data Availability

The datasets presented in this article are not readily available. The data is provided by Arkansas Center for Health Improvements. Requests to access the data should be submitted to this center (https://achi.net/).
