# Supplementary material for: Inequitable access to risk-appropriate neonatal care: evidence from a regionalized perinatal system in Arkansas
Source: Front Reprod Health. 2026 Aug 14;8:1914178. doi: 10.3389/frph.2026.1914178 (PMC13522216; doi:10.3389/frph.2026.1914178)
Supplement: Supplementary file 1 [file Datasheet1.docx]

Supplemental Material:

Inequitable Access to Risk-Appropriate Neonatal Care:

Evidence from a Regionalized Perinatal System in Arkansas

**1	Methods**

**Figure 1:** Overview of data sources, data preprocessing and statistical analyses.

Figure 1 summarizes the overall study design, illustrating the workflow from data acquisition and cleaning through final analysis.

**Data Sources and Study Population:** The data for this study were drawn from multiple sources, including the Arkansas All-Payer Claims Database (AR-APCD), the March of Dimes (MoD),^1^ and the Arkansas GIS Office.^2^ The AR-APCD is compiled at the state level by the Arkansas Center for Health Improvement (ACHI) and integrates statewide administrative and public health datasets from the Arkansas Department of Health. This dataset includes birth and death certificates, public and private health insurance claims, and other records. For this analysis, we used data derived from birth certificates. The MoD provides county-level maternity care access classification. The Arkansas GIS Office provides county boundary and spatial reference data.

Birth certificate records included all 358,183 live births occurring in Arkansas between 2014 and 2023. Of the total births, 356,017 (99.4%) were linked to county access data and used to analyze NICU utilization and neonatal transfer rate (the rest had county data missing). For triage and distance analyses, a cohort of 347,289 births was used after excluding records missing facility-level data.

**Hospital Registry and Triage Classification:** We constructed a comprehensive registry of all Arkansas birthing hospitals as well as major out-of-state referral centers. Facility neonatal levels of care (Levels I–IV) were taken from the Arkansas Department of Health’s perinatal regionalization list (2016) and cross-referenced against ACHI’s roster of birthing hospitals and the U.S. Maternal Health Portal; when a level was not explicit, we verified via hospital websites.^3–5^

For each birth, we determined the level of neonatal care required using infant clinical characteristics recorded on the birth certificate, including birth weight, gestational age, and the presence of major clinical complications.

1. Level IV: Assigned to infants with major surgical anomalies (e.g., gastroschisis, omphalocele, or congenital heart defects).
2. Level III: Assigned to very preterm infants ($<32$ weeks gestation), very-low-birth-weight (VLBW) infants ($<1,500$ g), or those requiring critical interventions such as prolonged ventilation, surfactant therapy, or treatment for seizures.
3. Level II: Assigned to moderate preterm infants (32–36 weeks), low-birth-weight (LBW) infants (1,500–2,499 g), or those requiring brief ventilation.
4. Level I: Assigned to all remaining newborns, typically term infants of normal birth weight requiring routine care.

We operationalized birth and interfacility transfer triage by comparing this required care level to the facility’s designated level. During birth, risk-appropriate care was defined as an exact match between infant need and birth facility level; over-triage occurred when care was provided at a higher level than required, and under-triage occurred when care was provided at a lower level. We applied the same classification framework to interfacility neonatal transfers. For each transfer from the delivery hospital, we compared the infant’s required level of care with the neonatal level of the receiving hospital to determine whether the transfer was risk-appropriate, over-triaged, or under-triaged.

**Geographic Access and Impact of Closures:** In addition to using March of Dimes data, which categorized counties according to maternity care access status, our analysis assessed access using straight-line (geodesic) distances;^6^ for triage at birth, we measured the distance from the mother’s home county centroid to the actual delivery facility and to the nearest facility capable of providing risk-appropriate care; for neonatal transfers, we calculated the distance from the birth facility to the actual receiving facility and compared it to the distance from the birth facility to the nearest risk-appropriate alternative . Additionally, local care availability was characterized by counting the number of lower-level, risk-appropriate, and higher-level hospitals within 50, 100, and 150-mile bands of the mother’s residence (for birth time facility availability), and of birth facility (for transferring facility availability). We obtained county boundary and centroid data from the Arkansas GIS Office to calculate these distances.^2^

We conducted a pre–post impact analysis of five obstetric unit closures (four rural Level I and one urban Level II). To quantify the specific travel burden imposed on families following these closures, we calculated the distance from the maternal home county centroid to the actual birth facility both before and after the closure month. Notably, for the transfer analysis associated with these closures, we measured the distance from the maternal home county to the receiving transfer facility—rather than from the birthing hospital—to accurately capture the cumulative travel burden on the maternal–infant dyad. Because two closures occurred late in 2023 (Ashley and Bradley counties), post-closure follow-up was insufficient for stable estimates; therefore, inferential analyses focus on counties with adequate observation periods.

We used Chi-square tests to compare categorical outcomes across multiple groups, such as transfer rates and NICU utilization across four levels of county maternity care access. Fisher’s exact tests were used for pairwise comparisons when sample sizes were small or expected cell counts were low (e.g., Medicaid vs. private insurance). To quantify the strength of associations, we reported Cramér’s V alongside Chi-square results. We calculated Wilson 95% confidence intervals for all estimated rates to characterize uncertainty. All statistical tests were two-sided. Statistical significance was defined as 𝛼 = 0.05 for overall Chi square tests. For pairwise comparisons, we applied Bonferroni correction by dividing 0.05 by the number of comparisons (e.g., 𝛼 = 0.05/6 = 0.0083 for pairwise comparisons across four county access categories). Differences in median travel distances were evaluated using Mann–Whitney U tests. To evaluate temporal trends in birth and transfer triage rates, we used the Kruskal–Wallis H test. Although annual statewide percentages provide a descriptive overview, they do not capture geographic variation or permit statistical comparison of distributions over time. Therefore, analyses were conducted at the county–year level and restricted to county–years with at least 50 births or at least 5 transfers to ensure stable estimates. The Kruskal–Wallis test, a nonparametric alternative to one-way ANOVA, was used to compare distributions across years without assuming normality. Statistical analyses were conducted using Python (version 3.7.6). Chi-square, Fisher’s exact, and Kruskal–Wallis tests were performed using SciPy, Wilson 95% confidence intervals were calculated using statsmodels, and Cramér’s V tests were computed using NumPy. Geographic distances were calculated using geopy’s geodesic function. Figures were generated using Matplotlib and Seabon.

# References

[1] March of Dimes. *Nowhere to Go: Maternity Care Deserts Across the US*. [https://www.marchofdimes.org/maternitycare-deserts-report.](https://www.marchofdimes.org/maternity-care-deserts-report) 2024.
[2] Arkansas GIS Office. *Arkansas County Boundaries*. [https://gis.arkansas.gov.](https://gis.arkansas.gov/) Shapefile. 2024.
[3] Arkansas Department of Health. *Perinatal Regionalization — Arkansas Maternal and Perinatal Outcomes Quality*

*Review Committee*. 2016. url: [https://healthy.arkansas.gov/boards-commissions/committees/arkansasmaternal-perinatal-outcomes-quality-review-committee/perinatal-regionalization/](https://healthy.arkansas.gov/boards-commissions/committees/arkansas-maternal-perinatal-outcomes-quality-review-committee/perinatal-regionalization/) (visited on

11/29/2025).

[4] Arkansas Center for Health Improvement. *Arkansas Birthing Hospitals*. 2025. url: [https://achi.net/maternalinfant-health/#arkansas-birthing-hospitals](https://achi.net/maternal-infant-health/#arkansas-birthing-hospitals) (visited on 11/29/2025).
[5] U.S. Maternal Health Portal. *Arkansas: Maternity Care Access*. 2025. url: [https://ar.maternalhealth.us/ maternity-care-access](https://ar.maternalhealth.us/maternity-care-access) (visited on 11/29/2025).
[6] M. E. Meredith, L. N. Steimle, and S. M. Radke. “The implications of using maternity care deserts to measure progress in access to obstetric care: a mixed-integer optimization analysis”. In: *BMC Health Services Research* 24.1 (May 2024), p. 682.
